# Tetra­quinolinium ditelluro(VI)octa­vanadate(V) octa­hydrate

**DOI:** 10.1107/S1600536813027347

**Published:** 2013-10-12

**Authors:** Sirine Toumi, Samah Akriche Toumi, Mohamed Rzaigui

**Affiliations:** a Laboratoire de chimie des Matériaux, Faculté des Sciences de Bizerte, 7021 Zarzouna Bizerte, Tunisia

## Abstract

In the title compound, (C_9_H_8_N)_4_[Te_2_V_8_O_28_]·8H_2_O, the com­plete heteropolyanion is generated by a crystallographic inversion centre. One of the two quniolinium ions forms an N—H⋯O_p_ (p = polyoxidometallate) hydrogen bond and the other an N—H⋯O_w_ (w = water) hydrogen bond. The water mol­ecules further link the components by O—H⋯O_p_ and O—H⋯O_w_ hydrogen bonds. A number of C—H⋯O inter­actions and aromatic π–π stacking inter­actions [shortest centroid–centroid separation = 3.541 (7) Å] are also observed. Together, these generate a three-dimensional network.

## Related literature

For applications of polyoxidometallates, see: Fukuda & Yamase (1997 ▶); Rajakumar *et al.* (2000 ▶); Folbergrova & Mares (1987 ▶); Fantus *et al.* (1995 ▶). For bond-valence calculations, see: Brown & Altermatt (1985 ▶). For geometrical features in related structures, see: Lee *et al.* (2008 ▶); Joo *et al.* (2011 ▶); Strukan *et al.* (1997 ▶); Konaka *et al.* (2008 ▶, 2011 ▶); Evans *et al.* (1966 ▶); Hemissi *et al.* (2010 ▶).
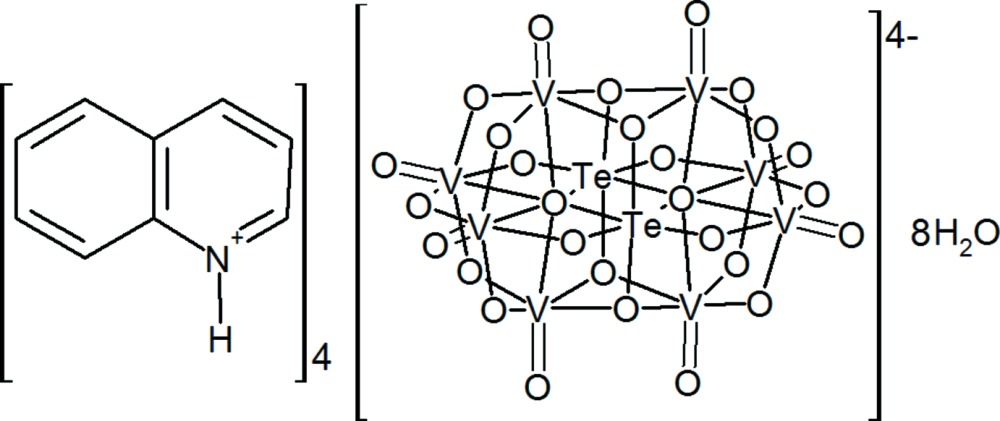


## Experimental

### 

#### Crystal data

(C_9_H_8_N)_4_[Te_2_V_8_O_28_]·8H_2_O
*M* *_r_* = 1775.50
Triclinic, 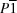

*a* = 10.907 (3) Å
*b* = 11.302 (3) Å
*c* = 13.169 (2) Å
α = 106.45 (4)°
β = 107.71 (4)°
γ = 105.34 (4)°
*V* = 1369.4 (6) Å^3^
*Z* = 1
Ag *K*α radiation
λ = 0.56087 Å
μ = 1.28 mm^−1^
*T* = 295 K
0.19 × 0.15 × 0.09 mm

#### Data collection

Enraf–Nonius CAD-4 diffractometer
Absorption correction: analytical (Alcock, 1970 ▶) *T*_min_ = 0.561, *T*_max_ = 0.725
16455 measured reflections
13245 independent reflections
6549 reflections with *I* > 2σ(*I*)
*R*_int_ = 0.045
2 standard reflections every 120 min intensity decay: 4%

#### Refinement

*R*[*F* ^2^ > 2σ(*F* ^2^)] = 0.115
*wR*(*F* ^2^) = 0.308
*S* = 1.05
13245 reflections
385 parameters
20 restraints
H atoms treated by a mixture of independent and constrained refinement
Δρ_max_ = 3.11 e Å^−3^
Δρ_min_ = −2.28 e Å^−3^

### 

Data collection: *CAD-4 EXPRESS* (Enraf–Nonius, 1994 ▶); cell refinement: *CAD-4 EXPRESS*; data reduction: *XCAD4* (Harms & Wocadlo, 1996 ▶); program(s) used to solve structure: *SHELXS97* (Sheldrick, 2008 ▶); program(s) used to refine structure: *SHELXL97* (Sheldrick, 2008 ▶); molecular graphics: *ORTEP-3 for Windows* (Farrugia, 2012 ▶) and *DIAMOND* (Brandenburg & Putz, 2005 ▶); software used to prepare material for publication: *WinGX* (Farrugia, 2012 ▶).

## Supplementary Material

Crystal structure: contains datablock(s) I. DOI: 10.1107/S1600536813027347/hb7146sup1.cif

Structure factors: contains datablock(s) I. DOI: 10.1107/S1600536813027347/hb7146Isup2.hkl

Additional supplementary materials:  crystallographic information; 3D view; checkCIF report

## Figures and Tables

**Table 1:** Hydrogen-bond geometry (Å, °)

*D*—H⋯*A*	*D*—H	H⋯*A*	*D*⋯*A*	*D*—H⋯*A*
O1*W*—H2*W*1⋯O2^i^	0.85 (1)	1.82 (1)	2.663 (8)	178 (1)
O1*W*—H1*W*1⋯O7	0.85 (1)	1.92 (2)	2.762 (9)	170 (4)
O2*W*—H1*W*2⋯O8^ii^	0.85 (1)	2.03 (2)	2.842 (10)	159 (4)
O2*W*—H2*W*2⋯O4*W* ^iii^	0.85 (1)	2.10 (1)	2.952 (14)	177 (1)
O3*W*—H2*W*3⋯O4*W*	0.84 (1)	1.91 (1)	2.754 (14)	177 (1)
O3*W*—H1*W*3⋯O6^ii^	0.85 (1)	1.83 (2)	2.665 (11)	166 (6)
O4*W*—H2*W*4⋯O1*W* ^iv^	0.85 (1)	2.41 (3)	2.826 (12)	111 (3)
O4*W*—H1*W*4⋯O2*W*	0.85 (1)	2.26 (5)	2.836 (17)	125 (5)
N1—H1⋯O1*W*	0.86	1.85	2.700 (11)	172
N2—H2⋯O14	0.86	1.88	2.740 (9)	175
C5—H5⋯O6^ii^	0.93	2.29	3.180 (13)	160
C6—H6⋯O13^v^	0.93	2.48	3.178 (14)	132
C7—H7⋯O1^vi^	0.93	2.56	3.350 (13)	143
C7—H7⋯O2*W* ^vii^	0.93	2.57	3.296 (14)	135
C10—H10⋯O9^viii^	0.93	2.51	3.275 (13)	140
C14—H14⋯O4^ix^	0.93	2.58	3.403 (14)	148
C17—H17⋯O5	0.93	2.60	3.411 (13)	146

Symmetry codes: (i) 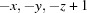; (ii) 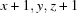; (iii) 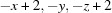; (iv) 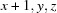; (v) 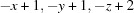; (vi) 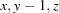; (vii) 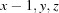; (viii) 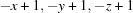; (ix) 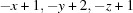.

**Fig. 1.**
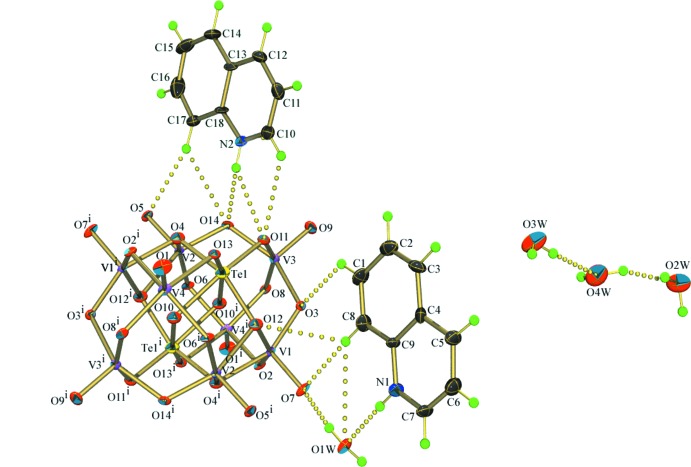
An ORTEP view of (I) with displacement ellipsoids drawn at the 30% probability level. Hydrogen bonds are represented as dashed lines. [Symmetry code: (i) 1 - x, 1 - y, - z]

**Fig. 2.**
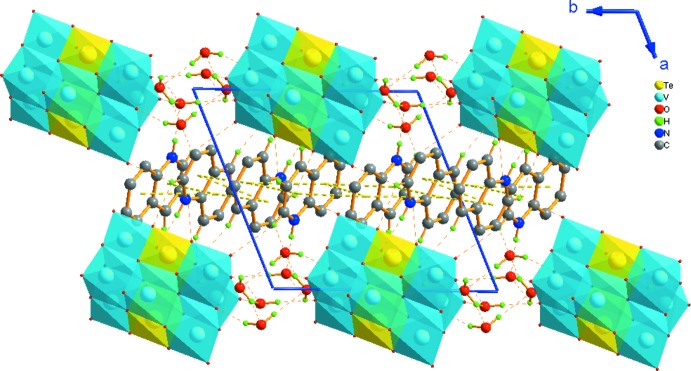
Structure projection of (I) along the [001] direction. The π ···π stacking interaction are represented as yellow dashed lines and the hydrogen bonds by red ones. The H-atoms not involved in H-bonding are omitted.

## supplementary crystallographic information

### 1. Comment

The polyoxovanadate materials (POVs) are well established in magnetic, electric and biomedical fields because of their richness structural variety in relation with the vanadium element which can adopt different coordination geometries and variable oxidation states very interesting for redox applications in catalysis and materials science (Fukuda *et al.*, 1997; Rajakumar *et al.*, 2000; Folbergrova *et al.*, 1987; Fantus *et al.*,1995). Furthermore, this family is dominated by the decavanadates, well known in their protonated forms [H*_x_*V_10_O_28_]^(6 -^*^x^*^)-^. Nevertheless, incorporation of other elements besides vanadium into this structural type has not been reported yet. In particular, substitution of one or more vanadium atoms by Pt^IV^ (Lee *et al.*, 2008; Joo *et al.*, 2011), Mo^VI^(Strukan *et al.*, 1997) and Te^VI^ (Konaka *et al.*, 2008; Konaka *et al.*, 2011) are investigated and resulted into novel materials with highly promising catalytic properties. To date in our knowledge, only two POVs incorporated one telluric, are known (Konaka *et al.*, 2008). Here, we describe the synthesis and structure of the first ocatavanado-ditellurate ion, [Te_2_V_8_O_28_]^4-^, which was isolated as the hydrated quinolinium salt (C_9_H_8_N)_4_[Te_2_V_8_O_28_]·8H_2_O (I). The asymmetric unit of (I) contains one half of a [Te_2_V_8_O_28_]^4-^ anion, two crystallographically independent quinolinium cations and four water molecules. Owing to the inversion symmetry, the whole polyanion is generated, resulting so to the title compound formulae (Fig. 1). The structure of the [Te_2_V_8_O_28_]^4-^ polyanion is basically the same as that of decavanadate, [V_10_O_28_]^6-^ (Evans *et al.*, 1966). In [Te_2_V_8_O_28_]^4-^ anion two of the central V atoms of [V_10_O_28_]^6-^ are replaced with two Te atoms. The valence bond calculation (Brown & Altermatt, 1985) gives effective bond valences of 6.1647 for Te cation and of 5.0743, 5.0068, 5.0574 and 5.063 for the four independent V cations, consistent with their oxidation states Te(+VI) and V(+V). Replacement of central V atom with a heteroatom, has also been observed for [H_2_PtV_9_O_28_]^5-^ (Lee *et al.*,2008; Joo *et al.*, 2011). As for the decavanadate anion, the [Te_2_V_8_O_28_]^4-^ anion is built up of 10 e dge sharing MO_6_ (*M* = V or Te) octahedra. Within VO_6_ octahedra, the V—O distances are also similar to those already observed and depend upon the type of oxo ligand: bond lengths to the terminal oxo oxygen V—Ot are between 1.602 (7) and 1.610 (6) Å, V—O2b bond lengths to the oxygen bonded to two V atoms vary from 1.767 (6) e t 1.864 (6) Å, V—O3b bond lengths to the oxygen bonded to three V atoms are 2.005 (7) and 2.039 (7) and finally, V—O6c bond lengths to the oxygen shared between six V atoms are 2.380 (7) and 2.387 (7) Å. The VO_6_ octahedra are significantly distorted, with the bond angles at the V atoms ranging from 73.5 (3) to 176.5 (3)°. The TeO_6_ octahedra are less distorted in comparison with VO_6_ ocathedra since the Te—O distances vary from 1.765 (6) to 2.080 (7) Å. Similar trends are also observable for similar heteropolyanion (Konaka *et al.*, 2008; Konaka *et al.*, 2011). The quinolinium cations exhibit the typical ranges in bond lengths and angles as found in the related structures (Hemissi *et al.*, 2010).

In the crystal packing, the discrete [Te_2_V_8_O_28_]^4-^ polyanions are hydrogen bonded through clusters of eight water molecules [H_2_O]_8_ forming layers [Te_2_V_8_O_28_(H_2_O)_8_]*_n_*^4^*^n^*^-^ stacked along the [100] direction (Fig. 2). With regard to the organic moieties, the two crystallographically independent [C_9_H_7_—N(1)H]^+^ and [C_9_H_7_—N(2)H]^+^ cations are interconnected thanks to intermolecular π ···π stacking interactions with centroid-centroid ring separations between 3.54 (8) and 3.90 (7) Å as to develop chains extending along [011] direction. Furthermore, the quinolinium chains and the polyanion sheets are linked thanks to O1W, O2W water molecules and terminal oxygen atoms (O1, O5 and O9) and bridged oxygen atoms (µ 2-O6, µ 2-O13, µ 2-O14 and µ 3-O4) of the polyanion, into a three dimensional network by N—H···O and C—H···O hydrogen bonds with donor—acceptor distances ranging from 2.700 (11) to 3.411 (13) Å.

### 2. Experimental

Vanadium (V) oxide (1.26 g, 6.93 mmol), quinoline (0.74 ml, 6.16 mmol) and telluric acid Te(OH)_6_ (0.36 g, 1.55 mmol) were dissolved in a mixture of 30 ml of distilled water and 10 ml of ethanol and then stirred for 3 h. Yellow single crystals were obtained after one week by slow evaporation at room temperature.

### 3. Refinement

All H atoms attached to C and N atoms were fixed geometrically and treated as riding, with C—H = 0.93 Å and N—H = 0.86 Å with *U*_iso_(H) = 1.2Ueq(C, N) for aromatic rings. The water H atoms were refined using restraints [O—H = 0.85 (1) A °, H···H = 1.44 (2) A ° and *U*_iso_(H) = 1.5Ueq(O)].

### Crystal data

**Table:**

(C_9_H_8_N)_4_[Te_2_V_8_O_28_]·8H_2_O	*Z* = 1
*M**_r_* = 1775.50	*F*(000) = 868
Triclinic, *P*1	*D*_x_ = 2.153 Mg m^−^^3^
Hall symbol: -P 1	Ag *K*α radiation, λ = 0.56087 Å
*a* = 10.907 (3) Å	Cell parameters from 25 reflections
*b* = 11.302 (3) Å	θ = 9–11°
*c* = 13.169 (2) Å	µ = 1.28 mm^−^^1^
α = 106.45 (4)°	*T* = 295 K
β = 107.71 (4)°	Rectangular, yellow
γ = 105.34 (4)°	0.19 × 0.15 × 0.09 mm
*V* = 1369.4 (6) Å^3^	

### Data collection

**Table:**

Enraf–Nonius CAD-4 diffractometer	6549 reflections with *I* > 2σ(*I*)
Radiation source: fine-focus sealed tube	*R*_int_ = 0.045
Graphite monochromator	θ_max_ = 28.0°, θ_min_ = 2.3°
non–profiled ω scans	*h* = −18→17
Absorption correction: analytical (Alcock, 1970)	*k* = −18→18
*T*_min_ = 0.561, *T*_max_ = 0.725	*l* = −3→22
16455 measured reflections	2 standard reflections every 120 min
13245 independent reflections	intensity decay: 4%

### Refinement

**Table:**

Refinement on *F*^2^	Primary atom site location: structure-invariant direct methods
Least-squares matrix: full	Secondary atom site location: difference Fourier map
*R*[*F*^2^ > 2σ(*F*^2^)] = 0.115	Hydrogen site location: inferred from neighbouring sites
*wR*(*F*^2^) = 0.308	H atoms treated by a mixture of independent and constrained refinement
*S* = 1.05	*w* = 1/[σ^2^(*F*_o_^2^) + (0.0978*P*)^2^ + 22.5177*P*] where *P* = (*F*_o_^2^ + 2*F*_c_^2^)/3
13245 reflections	(Δ/σ)_max_ < 0.001
385 parameters	Δρ_max_ = 3.11 e Å^−^^3^
20 restraints	Δρ_min_ = −2.28 e Å^−^^3^

### Special details

**Table:**

Geometry. All e.s.d.'s (except the e.s.d. in the dihedral angle between two l.s. planes) are estimated using the full covariance matrix. The cell e.s.d.'s are taken into account individually in the estimation of e.s.d.'s in distances, angles and torsion angles; correlations between e.s.d.'s in cell parameters are only used when they are defined by crystal symmetry. An approximate (isotropic) treatment of cell e.s.d.'s is used for estimating e.s.d.'s involving l.s. planes.
Refinement. Refinement of *F*^2^ against ALL reflections. The weighted *R*-factor *wR* and goodness of fit *S* are based on *F*^2^, conventional *R*-factors *R* are based on *F*, with *F* set to zero for negative *F*^2^. The threshold expression of *F*^2^ > σ(*F*^2^) is used only for calculating *R*-factors(gt) *etc*. and is not relevant to the choice of reflections for refinement. *R*-factors based on *F*^2^ are statistically about twice as large as those based on *F*, and *R*- factors based on ALL data will be even larger.

### Fractional atomic coordinates and isotropic or equivalent isotropic displacement parameters (Å^2^)

**Table:**

	*x*	*y*	*z*	*U*_iso_*/*U*_eq_	
Te1	0.16225 (7)	0.58886 (7)	0.58253 (6)	0.03140 (18)	
V1	0.05217 (14)	0.27420 (12)	0.49481 (12)	0.0177 (2)	
V2	0.03656 (13)	0.57033 (13)	0.32457 (11)	0.0166 (2)	
V3	0.23652 (14)	0.42319 (14)	0.39532 (13)	0.0213 (3)	
V4	0.07774 (16)	0.74944 (13)	0.76322 (12)	0.0215 (3)	
O1	0.1649 (8)	0.8702 (7)	0.8882 (6)	0.0356 (16)	
O2	−0.0552 (6)	0.1678 (5)	0.3401 (5)	0.0198 (10)	
O3	0.2094 (6)	0.3145 (6)	0.4763 (5)	0.0209 (11)	
O4	0.1186 (7)	0.6932 (7)	0.4925 (6)	0.03140 (18)	
O5	0.0293 (7)	0.6753 (6)	0.2649 (6)	0.0261 (12)	
O6	−0.0685 (6)	0.4051 (5)	0.2031 (5)	0.0209 (11)	
O7	0.0538 (8)	0.1673 (6)	0.5536 (6)	0.0289 (14)	
O8	0.1039 (6)	0.2887 (5)	0.2588 (5)	0.0208 (11)	
O9	0.3794 (7)	0.4249 (7)	0.3844 (7)	0.0322 (14)	
O10	−0.0326 (7)	0.5676 (7)	0.5778 (6)	0.03140 (18)	
O11	0.3065 (6)	0.5744 (6)	0.5518 (5)	0.0233 (11)	
O12	0.1318 (7)	0.4462 (7)	0.6351 (6)	0.03140 (18)	
O13	0.2328 (6)	0.7205 (6)	0.7204 (5)	0.0215 (11)	
O14	0.1984 (6)	0.5518 (6)	0.3384 (5)	0.0203 (10)	
O1W	0.0759 (8)	0.0847 (6)	0.7344 (6)	0.0335 (15)	
H1W1	0.067 (3)	0.118 (3)	0.683 (2)	0.040*	
H2W1	0.0671	0.0039	0.7102	0.040*	
O2W	1.1818 (11)	0.1180 (8)	1.1089 (9)	0.060 (3)	
H1W2	1.176 (13)	0.185 (2)	1.154 (12)	0.072*	
H2W2	1.1280	0.0421	1.0989	0.072*	
O3W	0.9613 (14)	0.3830 (8)	1.0059 (9)	0.065 (3)	
H1W3	0.947 (18)	0.401 (9)	1.068 (8)	0.078*	
H2W3	0.9696	0.3095	0.9801	0.078*	
O4W	0.9969 (13)	0.1478 (11)	0.9221 (9)	0.067 (3)	
H1W4	1.0634 (19)	0.121 (3)	0.936 (5)	0.081*	
H2W4	0.947 (3)	0.1236 (17)	0.8506 (12)	0.081*	
N1	0.3469 (8)	0.2174 (8)	0.8833 (7)	0.0351 (18)	
H1	0.2632	0.1746	0.8307	0.042*	
N2	0.4422 (8)	0.7675 (7)	0.4207 (8)	0.0297 (16)	
H2	0.3644	0.7027	0.3982	0.036*	
C1	0.4842 (14)	0.4522 (13)	0.7670 (11)	0.046 (3)	
H1A	0.4551	0.4753	0.7040	0.056*	
C2	0.6262 (15)	0.5203 (13)	0.8545 (13)	0.049 (3)	
H2A	0.6894	0.5873	0.8470	0.059*	
C3	0.6670 (12)	0.4882 (11)	0.9452 (13)	0.047 (3)	
H3	0.7581	0.5342	1.0011	0.056*	
C4	0.5747 (10)	0.3857 (10)	0.9581 (9)	0.0305 (18)	
C5	0.6157 (12)	0.3427 (12)	1.0502 (11)	0.047 (3)	
H5	0.7075	0.3808	1.1049	0.056*	
C6	0.5166 (14)	0.2435 (13)	1.0568 (11)	0.047 (3)	
H6	0.5410	0.2175	1.1186	0.056*	
C7	0.3861 (11)	0.1844 (11)	0.9754 (10)	0.039 (2)	
H7	0.3207	0.1189	0.9825	0.046*	
C8	0.3937 (12)	0.3527 (12)	0.7797 (10)	0.040 (2)	
H8	0.3017	0.3089	0.7251	0.048*	
C9	0.4371 (9)	0.3171 (9)	0.8717 (8)	0.0274 (17)	
C10	0.5429 (11)	0.7920 (10)	0.5154 (10)	0.036 (2)	
H10	0.5287	0.7373	0.5553	0.043*	
C11	0.6672 (12)	0.8923 (12)	0.5600 (12)	0.048 (3)	
H11	0.7361	0.9090	0.6306	0.057*	
C12	0.6909 (11)	0.9708 (11)	0.4981 (12)	0.043 (3)	
H12	0.7776	1.0383	0.5247	0.052*	
C13	0.5836 (10)	0.9467 (8)	0.3967 (11)	0.038 (2)	
C14	0.5994 (13)	1.0189 (12)	0.3266 (13)	0.049 (3)	
H14	0.6845	1.0867	0.3490	0.059*	
C15	0.4871 (19)	0.9884 (14)	0.2232 (16)	0.063 (4)	
H15	0.4970	1.0390	0.1793	0.076*	
C16	0.3654 (17)	0.8868 (15)	0.1870 (15)	0.066 (4)	
H16	0.2949	0.8661	0.1159	0.080*	
C17	0.3395 (11)	0.8109 (10)	0.2506 (11)	0.038 (2)	
H17	0.2524	0.7452	0.2267	0.046*	
C18	0.4533 (9)	0.8398 (8)	0.3537 (9)	0.0289 (18)	

### Atomic displacement parameters (Å^2^)

**Table:**

	*U*^11^	*U*^22^	*U*^33^	*U*^12^	*U*^13^	*U*^23^
Te1	0.0292 (3)	0.0266 (3)	0.0309 (3)	0.0036 (2)	0.0090 (2)	0.0112 (2)
V1	0.0244 (6)	0.0124 (5)	0.0195 (6)	0.0075 (4)	0.0102 (5)	0.0092 (5)
V2	0.0188 (5)	0.0153 (5)	0.0169 (6)	0.0046 (4)	0.0073 (5)	0.0100 (5)
V3	0.0195 (6)	0.0223 (6)	0.0271 (7)	0.0087 (5)	0.0126 (5)	0.0129 (5)
V4	0.0271 (7)	0.0146 (5)	0.0144 (6)	0.0031 (5)	0.0058 (5)	0.0018 (4)
O1	0.046 (4)	0.027 (3)	0.017 (3)	0.003 (3)	0.009 (3)	0.000 (2)
O2	0.027 (3)	0.012 (2)	0.019 (3)	0.0035 (19)	0.009 (2)	0.0077 (19)
O3	0.020 (2)	0.022 (3)	0.024 (3)	0.008 (2)	0.009 (2)	0.014 (2)
O4	0.0292 (3)	0.0266 (3)	0.0309 (3)	0.0036 (2)	0.0090 (2)	0.0112 (2)
O5	0.030 (3)	0.025 (3)	0.029 (3)	0.011 (2)	0.011 (3)	0.020 (3)
O6	0.023 (3)	0.011 (2)	0.017 (2)	−0.0030 (18)	0.004 (2)	0.0032 (18)
O7	0.050 (4)	0.018 (3)	0.023 (3)	0.014 (3)	0.016 (3)	0.013 (2)
O8	0.029 (3)	0.017 (2)	0.016 (2)	0.007 (2)	0.009 (2)	0.008 (2)
O9	0.027 (3)	0.042 (4)	0.040 (4)	0.017 (3)	0.022 (3)	0.022 (3)
O10	0.0292 (3)	0.0266 (3)	0.0309 (3)	0.0036 (2)	0.0090 (2)	0.0112 (2)
O11	0.018 (2)	0.025 (3)	0.024 (3)	0.003 (2)	0.008 (2)	0.011 (2)
O12	0.0292 (3)	0.0266 (3)	0.0309 (3)	0.0036 (2)	0.0090 (2)	0.0112 (2)
O13	0.017 (2)	0.019 (2)	0.022 (3)	0.0013 (19)	0.006 (2)	0.008 (2)
O14	0.017 (2)	0.023 (3)	0.019 (3)	0.0009 (19)	0.007 (2)	0.011 (2)
O1W	0.047 (4)	0.022 (3)	0.028 (3)	0.013 (3)	0.009 (3)	0.013 (3)
O2W	0.080 (7)	0.051 (5)	0.058 (6)	0.021 (5)	0.051 (6)	0.013 (5)
O3W	0.110 (9)	0.050 (5)	0.043 (5)	0.027 (6)	0.045 (6)	0.019 (4)
O4W	0.095 (8)	0.075 (7)	0.052 (6)	0.037 (6)	0.045 (6)	0.032 (5)
N1	0.029 (4)	0.030 (4)	0.031 (4)	0.001 (3)	0.001 (3)	0.013 (3)
N2	0.028 (3)	0.016 (3)	0.040 (4)	0.004 (3)	0.012 (3)	0.009 (3)
C1	0.063 (8)	0.053 (7)	0.041 (6)	0.026 (6)	0.027 (6)	0.033 (6)
C2	0.062 (8)	0.040 (6)	0.065 (8)	0.022 (6)	0.045 (7)	0.025 (6)
C3	0.028 (5)	0.033 (5)	0.067 (8)	0.000 (4)	0.021 (5)	0.013 (5)
C4	0.030 (4)	0.030 (4)	0.030 (5)	0.017 (4)	0.007 (4)	0.010 (4)
C5	0.030 (5)	0.042 (6)	0.050 (7)	0.000 (4)	−0.001 (5)	0.024 (5)
C6	0.051 (7)	0.046 (6)	0.043 (6)	0.017 (5)	0.012 (5)	0.025 (5)
C7	0.034 (5)	0.039 (5)	0.037 (5)	0.002 (4)	0.009 (4)	0.024 (4)
C8	0.040 (5)	0.046 (6)	0.028 (5)	0.014 (5)	0.007 (4)	0.016 (4)
C9	0.026 (4)	0.027 (4)	0.030 (4)	0.004 (3)	0.013 (3)	0.016 (3)
C10	0.037 (5)	0.030 (4)	0.043 (6)	0.015 (4)	0.020 (5)	0.012 (4)
C11	0.030 (5)	0.043 (6)	0.059 (8)	0.016 (5)	0.008 (5)	0.014 (6)
C12	0.026 (4)	0.029 (5)	0.065 (8)	0.003 (4)	0.019 (5)	0.013 (5)
C13	0.030 (4)	0.011 (3)	0.064 (7)	−0.001 (3)	0.021 (5)	0.008 (4)
C14	0.039 (6)	0.036 (5)	0.073 (9)	0.002 (4)	0.026 (6)	0.030 (6)
C15	0.098 (12)	0.043 (7)	0.092 (12)	0.033 (8)	0.075 (11)	0.042 (8)
C16	0.063 (9)	0.047 (7)	0.061 (9)	0.028 (7)	0.001 (7)	0.002 (7)
C17	0.034 (5)	0.025 (4)	0.052 (7)	0.006 (4)	0.017 (5)	0.018 (4)
C18	0.025 (4)	0.011 (3)	0.043 (5)	−0.003 (3)	0.018 (4)	0.005 (3)

### Geometric parameters (Å, º)

**Table:**

Te1—O13	1.765 (6)	O12—V2^i^	2.039 (7)
Te1—O11	1.775 (6)	O1W—H1W1	0.853 (10)
Te1—O12	1.918 (7)	O1W—H2W1	0.845 (5)
Te1—O4	1.941 (7)	O2W—H1W2	0.849 (10)
Te1—O10	2.054 (7)	O2W—H2W2	0.849 (7)
Te1—O10^i^	2.080 (7)	O3W—H1W3	0.850 (10)
Te1—V4	3.095 (2)	O3W—H2W3	0.844 (7)
Te1—V3	3.105 (2)	O4W—H1W4	0.850 (10)
Te1—V1	3.161 (2)	O4W—H2W4	0.849 (10)
Te1—V2	3.1740 (18)	N1—C7	1.348 (13)
Te1—Te1^i^	3.220 (3)	N1—C9	1.366 (11)
V1—O7	1.610 (6)	N1—H1	0.8600
V1—O3	1.764 (6)	N2—C10	1.283 (14)
V1—O2	1.840 (6)	N2—C18	1.372 (12)
V1—O12	2.010 (7)	N2—H2	0.8600
V1—O4^i^	2.037 (7)	C1—C8	1.373 (17)
V1—O10^i^	2.281 (7)	C1—C2	1.45 (2)
V1—V2^i^	3.101 (2)	C1—H1A	0.9300
V1—V3	3.107 (2)	C2—C3	1.328 (19)
V2—O5	1.603 (6)	C2—H2A	0.9300
V2—O14	1.793 (6)	C3—C4	1.408 (15)
V2—O6	1.849 (6)	C3—H3	0.9300
V2—O4	2.005 (7)	C4—C9	1.412 (13)
V2—O12^i^	2.039 (7)	C4—C5	1.423 (15)
V2—O10^i^	2.285 (7)	C5—C6	1.380 (17)
V2—V1^i^	3.101 (2)	C5—H5	0.9300
V3—O9	1.604 (7)	C6—C7	1.336 (16)
V3—O8	1.828 (6)	C6—H6	0.9300
V3—O3	1.864 (6)	C7—H7	0.9300
V3—O14	1.895 (6)	C8—C9	1.372 (14)
V3—O11	2.027 (7)	C8—H8	0.9300
V3—O10^i^	2.380 (7)	C10—C11	1.339 (16)
V3—V4^i^	3.117 (3)	C10—H10	0.9300
V4—O1	1.602 (7)	C11—C12	1.390 (19)
V4—O8^i^	1.822 (6)	C11—H11	0.9300
V4—O2^i^	1.851 (6)	C12—C13	1.379 (18)
V4—O6^i^	1.905 (6)	C12—H12	0.9300
V4—O13	2.013 (6)	C13—C14	1.413 (17)
V4—O10	2.387 (7)	C13—C18	1.420 (12)
V4—V3^i^	3.117 (3)	C14—C15	1.40 (2)
O2—V4^i^	1.851 (6)	C14—H14	0.9300
O4—V1^i^	2.037 (7)	C15—C16	1.34 (2)
O6—V4^i^	1.905 (6)	C15—H15	0.9300
O8—V4^i^	1.822 (6)	C16—C17	1.39 (2)
O10—Te1^i^	2.080 (7)	C16—H16	0.9300
O10—V1^i^	2.281 (7)	C17—C18	1.414 (16)
O10—V2^i^	2.285 (7)	C17—H17	0.9300
O10—V3^i^	2.380 (7)		
			
O13—Te1—O11	106.2 (3)	O11—V3—Te1	32.64 (17)
O13—Te1—O12	96.4 (3)	O10^i^—V3—Te1	42.03 (17)
O11—Te1—O12	96.9 (3)	O9—V3—V1	133.6 (3)
O13—Te1—O4	96.4 (3)	O8—V3—V1	81.44 (19)
O11—Te1—O4	96.4 (3)	O3—V3—V1	30.14 (18)
O12—Te1—O4	158.2 (3)	O14—V3—V1	122.84 (18)
O13—Te1—O10	88.1 (3)	O11—V3—V1	82.33 (18)
O11—Te1—O10	165.7 (3)	O10^i^—V3—V1	46.83 (17)
O12—Te1—O10	81.5 (3)	Te1—V3—V1	61.17 (5)
O4—Te1—O10	81.3 (3)	O9—V3—V4^i^	134.2 (3)
O13—Te1—O10^i^	165.8 (3)	O8—V3—V4^i^	31.3 (2)
O11—Te1—O10^i^	88.0 (3)	O3—V3—V4^i^	83.04 (19)
O12—Te1—O10^i^	81.8 (3)	O14—V3—V4^i^	83.61 (18)
O4—Te1—O10^i^	81.4 (3)	O11—V3—V4^i^	123.93 (18)
O10—Te1—O10^i^	77.7 (3)	O10^i^—V3—V4^i^	49.27 (17)
O13—Te1—V4	37.73 (19)	Te1—V3—V4^i^	91.29 (7)
O11—Te1—V4	143.9 (2)	V1—V3—V4^i^	61.01 (5)
O12—Te1—V4	90.4 (2)	O1—V4—O8^i^	104.6 (3)
O4—Te1—V4	89.0 (2)	O1—V4—O2^i^	104.4 (3)
O10—Te1—V4	50.4 (2)	O8^i^—V4—O2^i^	89.8 (3)
O10^i^—Te1—V4	128.1 (2)	O1—V4—O6^i^	103.4 (3)
O13—Te1—V3	144.19 (19)	O8^i^—V4—O6^i^	88.9 (3)
O11—Te1—V3	38.0 (2)	O2^i^—V4—O6^i^	151.5 (3)
O12—Te1—V3	89.5 (2)	O1—V4—O13	100.9 (3)
O4—Te1—V3	90.3 (2)	O8^i^—V4—O13	154.4 (3)
O10—Te1—V3	127.7 (2)	O2^i^—V4—O13	85.6 (3)
O10^i^—Te1—V3	50.0 (2)	O6^i^—V4—O13	83.4 (3)
V4—Te1—V3	178.06 (5)	O1—V4—O10	174.8 (4)
O13—Te1—V1	133.8 (2)	O8^i^—V4—O10	80.5 (3)
O11—Te1—V1	84.6 (2)	O2^i^—V4—O10	76.5 (2)
O12—Te1—V1	37.4 (2)	O6^i^—V4—O10	75.3 (2)
O4—Te1—V1	127.5 (2)	O13—V4—O10	74.0 (2)
O10—Te1—V1	85.7 (2)	O1—V4—Te1	133.3 (3)
O10^i^—Te1—V1	46.1 (2)	O8^i^—V4—Te1	122.0 (2)
V4—Te1—V1	119.73 (6)	O2^i^—V4—Te1	78.91 (19)
V3—Te1—V1	59.45 (5)	O6^i^—V4—Te1	77.73 (19)
O13—Te1—V2	133.6 (2)	O13—V4—Te1	32.45 (17)
O11—Te1—V2	84.3 (2)	O10—V4—Te1	41.54 (17)
O12—Te1—V2	127.7 (2)	O1—V4—V3^i^	136.0 (3)
O4—Te1—V2	37.1 (2)	O8^i^—V4—V3^i^	31.41 (18)
O10—Te1—V2	85.5 (2)	O2^i^—V4—V3^i^	81.97 (19)
O10^i^—Te1—V2	45.95 (19)	O6^i^—V4—V3^i^	82.23 (18)
V4—Te1—V2	118.35 (5)	O13—V4—V3^i^	123.07 (18)
V3—Te1—V2	60.30 (5)	O10—V4—V3^i^	49.08 (18)
V1—Te1—V2	91.45 (7)	Te1—V4—V3^i^	90.62 (7)
O13—Te1—Te1^i^	127.3 (2)	V1—O2—V4^i^	117.8 (3)
O11—Te1—Te1^i^	126.5 (2)	V1—O3—V3	117.8 (3)
O12—Te1—Te1^i^	79.3 (2)	Te1—O4—V2	107.1 (3)
O4—Te1—Te1^i^	78.9 (2)	Te1—O4—V1^i^	107.2 (3)
O10—Te1—Te1^i^	39.14 (19)	V2—O4—V1^i^	100.2 (3)
O10^i^—Te1—Te1^i^	38.5 (2)	V2—O6—V4^i^	117.7 (3)
V4—Te1—Te1^i^	89.55 (6)	V4^i^—O8—V3	117.3 (3)
V3—Te1—Te1^i^	88.53 (6)	Te1—O10—Te1^i^	102.3 (3)
V1—Te1—Te1^i^	60.25 (6)	Te1—O10—V1^i^	95.2 (3)
V2—Te1—Te1^i^	60.03 (5)	Te1^i^—O10—V1^i^	92.8 (3)
O7—V1—O3	105.3 (3)	Te1—O10—V2^i^	94.9 (3)
O7—V1—O2	102.9 (3)	Te1^i^—O10—V2^i^	93.2 (3)
O3—V1—O2	93.8 (3)	V1^i^—O10—V2^i^	167.0 (4)
O7—V1—O12	101.4 (3)	Te1—O10—V3^i^	169.7 (4)
O3—V1—O12	91.1 (3)	Te1^i^—O10—V3^i^	88.0 (2)
O2—V1—O12	152.9 (3)	V1^i^—O10—V3^i^	83.6 (2)
O7—V1—O4^i^	99.3 (3)	V2^i^—O10—V3^i^	85.1 (2)
O3—V1—O4^i^	154.0 (3)	Te1—O10—V4	88.0 (2)
O2—V1—O4^i^	88.6 (3)	Te1^i^—O10—V4	169.6 (4)
O12—V1—O4^i^	75.9 (3)	V1^i^—O10—V4	85.2 (2)
O7—V1—O10^i^	172.9 (3)	V2^i^—O10—V4	86.9 (2)
O3—V1—O10^i^	81.1 (3)	V3^i^—O10—V4	81.7 (2)
O2—V1—O10^i^	79.5 (2)	Te1—O11—V3	109.4 (3)
O12—V1—O10^i^	75.0 (3)	Te1—O12—V1	107.1 (3)
O4^i^—V1—O10^i^	74.0 (3)	Te1—O12—V2^i^	107.8 (3)
O7—V1—V2^i^	89.7 (2)	V1—O12—V2^i^	99.9 (3)
O3—V1—V2^i^	131.4 (2)	Te1—O13—V4	109.8 (3)
O2—V1—V2^i^	128.1 (2)	V2—O14—V3	117.5 (3)
O12—V1—V2^i^	40.4 (2)	H1W1—O1W—H2W1	115 (3)
O4^i^—V1—V2^i^	39.5 (2)	H1W2—O2W—H2W2	115 (3)
O10^i^—V1—V2^i^	83.69 (19)	H1W3—O3W—H2W3	117 (3)
O7—V1—V3	137.1 (3)	H1W4—O4W—H2W4	115 (3)
O3—V1—V3	32.04 (18)	C7—N1—C9	121.5 (8)
O2—V1—V3	82.40 (18)	C7—N1—H1	119.2
O12—V1—V3	87.9 (2)	C9—N1—H1	119.2
O4^i^—V1—V3	123.5 (2)	C10—N2—C18	123.0 (8)
O10^i^—V1—V3	49.57 (18)	C10—N2—H2	118.5
V2^i^—V1—V3	120.78 (6)	C18—N2—H2	118.5
O7—V1—Te1	136.4 (2)	C8—C1—C2	118.3 (11)
O3—V1—Te1	77.3 (2)	C8—C1—H1A	120.9
O2—V1—Te1	120.50 (18)	C2—C1—H1A	120.9
O12—V1—Te1	35.4 (2)	C3—C2—C1	120.7 (11)
O4^i^—V1—Te1	79.20 (19)	C3—C2—H2A	119.6
O10^i^—V1—Te1	41.10 (17)	C1—C2—H2A	119.6
V2^i^—V1—Te1	61.43 (5)	C2—C3—C4	121.1 (11)
V3—V1—Te1	59.39 (5)	C2—C3—H3	119.4
O5—V2—O14	105.6 (3)	C4—C3—H3	119.4
O5—V2—O6	104.4 (3)	C3—C4—C9	118.3 (10)
O14—V2—O6	93.3 (3)	C3—C4—C5	123.2 (10)
O5—V2—O4	100.4 (3)	C9—C4—C5	118.5 (9)
O14—V2—O4	91.4 (3)	C6—C5—C4	118.8 (10)
O6—V2—O4	152.5 (3)	C6—C5—H5	120.6
O5—V2—O12^i^	100.0 (3)	C4—C5—H5	120.6
O14—V2—O12^i^	153.2 (3)	C7—C6—C5	120.7 (11)
O6—V2—O12^i^	88.1 (3)	C7—C6—H6	119.6
O4—V2—O12^i^	76.0 (3)	C5—C6—H6	119.6
O5—V2—O10^i^	172.8 (3)	C6—C7—N1	121.6 (10)
O14—V2—O10^i^	80.5 (2)	C6—C7—H7	119.2
O6—V2—O10^i^	78.9 (2)	N1—C7—H7	119.2
O4—V2—O10^i^	75.1 (3)	C9—C8—C1	120.9 (11)
O12^i^—V2—O10^i^	73.5 (3)	C9—C8—H8	119.6
O5—V2—V1^i^	89.4 (3)	C1—C8—H8	119.6
O14—V2—V1^i^	131.6 (2)	N1—C9—C8	120.7 (9)
O6—V2—V1^i^	127.8 (2)	N1—C9—C4	118.7 (8)
O4—V2—V1^i^	40.3 (2)	C8—C9—C4	120.6 (9)
O12^i^—V2—V1^i^	39.7 (2)	N2—C10—C11	123.5 (11)
O10^i^—V2—V1^i^	83.49 (19)	N2—C10—H10	118.2
O5—V2—Te1	135.7 (3)	C11—C10—H10	118.2
O14—V2—Te1	77.17 (19)	C10—C11—C12	118.4 (12)
O6—V2—Te1	119.75 (19)	C10—C11—H11	120.8
O4—V2—Te1	35.8 (2)	C12—C11—H11	120.8
O12^i^—V2—Te1	78.9 (2)	C13—C12—C11	118.9 (10)
O10^i^—V2—Te1	40.88 (18)	C13—C12—H12	120.6
V1^i^—V2—Te1	61.38 (5)	C11—C12—H12	120.6
O9—V3—O8	102.9 (3)	C12—C13—C14	122.8 (9)
O9—V3—O3	103.6 (3)	C12—C13—C18	120.4 (10)
O8—V3—O3	91.2 (3)	C14—C13—C18	116.7 (11)
O9—V3—O14	103.5 (3)	C15—C14—C13	120.2 (10)
O8—V3—O14	90.2 (2)	C15—C14—H14	119.9
O3—V3—O14	151.8 (3)	C13—C14—H14	119.9
O9—V3—O11	101.9 (3)	C16—C15—C14	120.6 (13)
O8—V3—O11	155.2 (3)	C16—C15—H15	119.7
O3—V3—O11	84.1 (3)	C14—C15—H15	119.7
O14—V3—O11	83.0 (3)	C15—C16—C17	123.3 (14)
O9—V3—O10^i^	176.5 (3)	C15—C16—H16	118.3
O8—V3—O10^i^	80.6 (3)	C17—C16—H16	118.3
O3—V3—O10^i^	76.5 (2)	C16—C17—C18	116.1 (10)
O14—V3—O10^i^	76.0 (2)	C16—C17—H17	122.0
O11—V3—O10^i^	74.7 (2)	C18—C17—H17	122.0
O9—V3—Te1	134.5 (3)	N2—C18—C17	121.4 (8)
O8—V3—Te1	122.6 (2)	N2—C18—C13	115.7 (10)
O3—V3—Te1	77.75 (19)	C17—C18—C13	122.9 (9)
O14—V3—Te1	77.87 (18)		
			
C8—C1—C2—C3	−0.8 (19)	C18—N2—C10—C11	1.8 (17)
C1—C2—C3—C4	1.2 (19)	N2—C10—C11—C12	−2.8 (18)
C2—C3—C4—C9	0.2 (17)	C10—C11—C12—C13	3.2 (18)
C2—C3—C4—C5	176.4 (12)	C11—C12—C13—C14	−178.1 (12)
C3—C4—C5—C6	178.5 (12)	C11—C12—C13—C18	−2.8 (17)
C9—C4—C5—C6	−5.3 (18)	C12—C13—C14—C15	178.9 (12)
C4—C5—C6—C7	3 (2)	C18—C13—C14—C15	3.4 (18)
C5—C6—C7—N1	1 (2)	C13—C14—C15—C16	−3 (2)
C9—N1—C7—C6	−3.1 (18)	C14—C15—C16—C17	4 (2)
C2—C1—C8—C9	−1.1 (19)	C15—C16—C17—C18	−5 (2)
C7—N1—C9—C8	−177.4 (11)	C10—N2—C18—C17	−179.1 (10)
C7—N1—C9—C4	0.7 (16)	C10—N2—C18—C13	−1.1 (14)
C1—C8—C9—N1	−179.5 (11)	C16—C17—C18—N2	−176.9 (10)
C1—C8—C9—C4	2.5 (17)	C16—C17—C18—C13	5.2 (16)
C3—C4—C9—N1	179.9 (10)	C12—C13—C18—N2	1.7 (14)
C5—C4—C9—N1	3.5 (15)	C14—C13—C18—N2	177.3 (10)
C3—C4—C9—C8	−2.0 (15)	C12—C13—C18—C17	179.7 (10)
C5—C4—C9—C8	−178.4 (11)	C14—C13—C18—C17	−4.7 (15)

Symmetry code: (i) −*x*, −*y*+1, −*z*+1.

### Hydrogen-bond geometry (Å, º)

**Table:**

*D*—H···*A*	*D*—H	H···*A*	*D*···*A*	*D*—H···*A*
O1*W*—H2*W*1···O2^ii^	0.85 (1)	1.82 (1)	2.663 (8)	178 (1)
O1*W*—H1*W*1···O7	0.85 (1)	1.92 (2)	2.762 (9)	170 (4)
O2*W*—H1*W*2···O8^iii^	0.85 (1)	2.03 (2)	2.842 (10)	159 (4)
O2*W*—H2*W*2···O4*W*^iv^	0.85 (1)	2.10 (1)	2.952 (14)	177 (1)
O3*W*—H2*W*3···O4*W*	0.84 (1)	1.91 (1)	2.754 (14)	177 (1)
O3*W*—H1*W*3···O6^iii^	0.85 (1)	1.83 (2)	2.665 (11)	166 (6)
O4*W*—H2*W*4···O1*W*^v^	0.85 (1)	2.41 (3)	2.826 (12)	111 (3)
O4*W*—H1*W*4···O2*W*	0.85 (1)	2.26 (5)	2.836 (17)	125 (5)
N1—H1···O1*W*	0.86	1.85	2.700 (11)	172
N2—H2···O14	0.86	1.88	2.740 (9)	175
C5—H5···O6^iii^	0.93	2.29	3.180 (13)	160
C6—H6···O13^vi^	0.93	2.48	3.178 (14)	132
C7—H7···O1^vii^	0.93	2.56	3.350 (13)	143
C7—H7···O2*W*^viii^	0.93	2.57	3.296 (14)	135
C10—H10···O9^ix^	0.93	2.51	3.275 (13)	140
C14—H14···O4^x^	0.93	2.58	3.403 (14)	148
C17—H17···O5	0.93	2.60	3.411 (13)	146

Symmetry codes: (ii) −*x*, −*y*, −*z*+1; (iii) *x*+1, *y*, *z*+1; (iv) −*x*+2, −*y*, −*z*+2; (v) *x*+1, *y*, *z*; (vi) −*x*+1, −*y*+1, −*z*+2; (vii) *x*, *y*−1, *z*; (viii) *x*−1, *y*, *z*; (ix) −*x*+1, −*y*+1, −*z*+1; (x) −*x*+1, −*y*+2, −*z*+1.
